# Fostering inclusive co-creation in digital health

**DOI:** 10.1038/s41746-025-01724-w

**Published:** 2025-05-26

**Authors:** Alessandro Blasimme, Constantin Landers, Effy Vayena

**Affiliations:** Department of Health Sciences and Technology, ETH Zurich, Zurich, Switzerland

**Keywords:** Health policy, Health care

## Abstract

Research on responsible digital health innovation has typically focused on technical aspects such as the reliability and trustworthiness. More recently, work in responsible digital health innovation has started to recognize that, to address those concerns, stakeholder involvement is key. Aligning technological advancements with stakeholders’ needs requires deliberate and inclusive processes. Such processes must incorporate diverse perspectives, including those of the users of digital health technologies, such as healthcare practitionerss and patients.

Giving due consideration to the societal, ethical and regulatory impact of technological change is crucial. Responsible innovation is therefore an ineludible element in a transformative field like digital health^1^. So far, research on responsible digital health innovation has typically focused on technical aspects such as accountability, reliability and trustworthiness^2,3^. In particular, considerable attention is drawn to the identification and mitigation of very well-known risks in digital health and artificial intelligence (AI) applications. Concerns include privacy and data protection risks, liability, transparency, explainability and mitigation of algorithmic biases.

More recently, work in responsible digital health innovation has started to recognize that, to address those concerns, stakeholder involvement is key^4^.

The notion of co-creation refers to technological development that “involves a broad range of relevant and affected actors in creative problem-solving that aims to produce a desired outcome”^5^. Different stakeholders can and should indeed contribute to the creation of digital health innovation, ensuring their interests and values can inform the design and characteristics of digital technologies for human health^4,5^. Nickel and colleagues^6^, for example, have recently drawn attention to the role of inclusive co-creation as one major determinant of responsible digital health innovation.

In a recent study published in this journal^7^, we have involved 46 healthcare experts and leaders who identified co-creation as one of the key components of responsible digital health innovation, alongside responsive regulation and value-driven innovation. Nickel and colleagues^6^ productively engage with our article’s findings, pointing out, however, that our stakeholders sample did not include healthcare practitioners (HCPs). We agree that HCPs are a key stakeholder group that should have ideally been included in our empirical study, and we have explicitly recognized their absence as one of the limitations of our work. Contingent factors have influenced this outcome. We started our study in a time when the COVID19 pandemic was still occupying much of HCPs’ time. Although we resorted to an original online stakeholder engagement methodology^7,8^ to recruit a broad array of stakeholders, HCPs showed limited interest in participating. Yet, six of the study participants were fully trained doctors, and some among them actively practice medicine despite participating in the study in other capacities, such as business leaders or regulatory affairs experts.

We would further like to stress that the categorization of HCPs as “Implementers & end-users” rather than “Innovators” does not represent a methodological choice by the authors, but reflects the views of participants, who first introduced it and then collectively endorsed it.

The challenge encountered in recruiting HCPs for our study may also indicate that insufficient motivation or inadequate incentives could potentially constrain their role in co-creation. On the other hand, it should be noted that stakeholders involved in our study could have stressed more clearly the importance of HCPs in co-creating responsible digital health, but actually didn’t. Nickel and colleagues thus rightly warn against the risk that other stakeholders play a dominant role in shaping development and implementation best practices in the digital health space, and that the role of HCPs is underestimated.

Based on the initial insights of our stakeholder engagement exercise, we have developed a roadmap containing specific recommendations on how decision-makers and key stakeholders can promote responsible digital health innovation^9^. While this roadmap is tailored to the Swiss healthcare ecosystem and its regulatory specificities, it contains nonetheless elements that are largely generalizable to other contexts.

Our roadmap explicitly emphasizes the role of HCPs as key stakeholders. More specifically, we envisage a key role for healthcare specialists in testing, monitoring, and customizing digital health solutions for the benefit of patients and to make healthcare provision more effective and inclusive^10^.

As regulation evolves around digital health applications and AI, more attention should be paid to the processes through which digital health tools are developed and implemented. HCPs can and should play a key role in proposing and testing best practices for the responsible implementation of digital innovation in the healthcare space. Participatory design and co-development are established methodologies, especially in fields like human-computer interaction^11,12^, with interest growing also in the field of digital health^13^. It remains to be seen, however, how HCPs can be meaningfully engaged as co-creators. We suggest that agile regulation can offer a blueprint to allow HCPs and other stakeholders to safely test and co-create new digital health solutions^9^. Agile regulation comprises mechanisms like sandboxing to test pilot new technologies as well as new approaches to their governance. Regulatory sandboxes allow stakeholders that would normally be left at the margins of digital health development and regulation to have their voice heard and exert a concrete influence on how technologies are created, tested, and validated for clinical use^14^. New modes of marketing authorization, product oversight and outcome monitoring can also be spearheaded thanks to regulatory experimentation, eventually indicating new routes for the effective and inclusive governance of digital health innovation^15^.

The processes and norms that shape digital health should arguably take the point of view of HCPs into account. Both as mediators of the digital transformation of healthcare and as end-users of digital health products, HCPs are not only useful in shaping innovation, but also bear a responsibility to help steer it in the best interest of their patients and of healthcare systems more generally^10^. HCPs, however, cannot fulfill such responsibility just out of their own initiative. Digital health innovation should instead be understood as an innovation ecosystem enabling dialogue and cooperation among different stakeholder groups that understand each other as co-creators. Companies, policy- makers, and regulators in the digital health space possess considerable leverage in enabling the involvement of key stakeholder groups, such as HCPs and patients. In particular, such stakeholders can contribute with their lived experience, needs, and preferences to the design and development of more effective, inclusive, and responsible innovations. By creating the legal conditions for agile regulation, policy-makers can thus enable co-creation with the aim of aligning innovation around stakeholders’ needs and expectations. Clearly, the effectiveness and inclusivity of co-creation practices depends also on addressing background conditions that affect stakeholders’ ability to be meaningfully engaged in co-creation, including issues of equity and structural injustice^16^.

The high pace of digital innovation fuels both expectations and concerns about the impact of transformative technologies such as artificial intelligence (AI) in the domain of healthcare. Such transformation and its associated challenges are not mere technical issues that can be addressed by developers and engineers alone. Aligning technological advancements with stakeholders’ needs requires deliberate and inclusive processes. Such processes must incorporate diverse perspectives, including the users of digital health technologies, such as HCPs and patients.

## Data Availability

No datasets were generated or analysed during the current study.
